# Anonymous Online Survey on Disordered Eating, Drive for Muscularity, Sexual Orientation, and Satisfaction with Life in Young Swedish Males

**DOI:** 10.1007/s10508-022-02383-8

**Published:** 2022-08-16

**Authors:** Ata Ghaderi, Cynthia Bulik, Mattias Myrälf, Elisabeth Welch

**Affiliations:** 1 Division of Psychology, Department of Clinical Neuroscience, Karolinska Institutet, 17177 Stockholm, Sweden; 2 Stockholm Health Care Services, Region Stockholm, Stockholm Centre for Eating Disorders, Stockholm, Sweden; 3 Department of Psychiatry, University of North Carolina at Chapel Hill, Chapel Hill, USA; 4 Department of Nutrition, University of North Carolina at Chapel Hill, Chapel Hill, USA; 5 Department of Medical Epidemiology and Biostatistics, Karolinska Institutet, Stockholm, Sweden; 6 Department of Women’s and Children’s Health, Uppsala University, Uppsala, Sweden; 7 Department of Clinical Neuroscience, Centre for Psychiatry Research, Karolinska Institutet, Stockholm, Sweden

**Keywords:** Males, Drive for muscularity, Eating disorders, Sexual preference, Anonymous survey

## Abstract

Psychiatric conditions in general, including eating disorders, are stigmatizing conditions. The stigma of eating disorders is even more pronounced among males. We conducted an anonymous, online survey to explore the feasibility of recruiting participants for collecting sensitive information, and the relation among eating disorders, drive for muscularity, satisfaction with life, and sexual preference in males (*N* = 824) aged 15–30 years in Sweden. Internet survey method was a feasible way of recruiting males and obtaining sensitive information. Drive for muscularity was positively related to eating psychopathology. Interestingly, only the attitudinal aspect of the drive for muscularity was negatively related to satisfaction with life, whereas the behavioral component of the drive for muscularity was unrelated to quality of life. Drive for muscularity and disordered eating were not significantly different across participants with various sexual orientations. Our findings corroborate and extend previous research by using an anonymous Internet-based survey that may be less contaminated by social desirability or reporting bias due to the sensitivity of some of the questions.

## Introduction

Eating disorders (ED) and disordered eating behavior and attitudes are of considerable clinical and public health concern as they are prevalent and are associated with negative health consequences, functional impairment, high comorbidity, and impaired quality of life (Arcelus et al., 2011; Stice et al., 2013). Eating disorders are also stigmatizing conditions (Ali et al., 2020; Griffiths et al., 2014; Guy et al., 2021; Lupo et al., 2020). In Western society, disordered eating attitudes and behaviors, as well as body image concerns, are typically attributed to female gender (Jones & Morgan, 2010). However, several studies suggest that these problems are prevalent in males as well (Field et al., 2014; Hautala et al., 2008; O'Dea & Abraham, 2002). In a large study of 3890 Swiss males, Dominé et al. (2009) reported that about half of adolescent boys aged 16–20 were affected by some kind of eating concern or unhealthy eating behavior. The number of studies on symptoms and correlates of ED among men has been increasing during the last two decades (Carper et al., 2010; Griffiths et al., 2013; Kaminski et al., 2005; Smith et al., 2011; Yean et al., 2013). However, some of the questions about ED and other topics (e.g., sexual orientation) might be perceived as sensitive and embarrassing, and may lead to response bias or non-participation. Misreporting about sensitive topics is common and it seems to be largely situational (Tourangeau & Yan, 2007). Therefore, efficient ways of recruiting males to participate in studies about ED that minimize the risk of response bias or non-participation are needed. Anonymous Internet-based surveys might provide such a solution. Web-based survey formats may improve rates of self-disclosure, especially in young college-aged males being queried about topics that may be perceived as highly sensitive (Kays et al., 2012). Efficient recruitment of men into studies designed to gather reliable information may assist with clarifying the nature of EDs in males.

Much of the work on the phenomenology of eating disorders has come from research on girls and women. The work that has been conducted on boys and men suggests that eating disorders and disordered eating behavior may be expressed differently across the sexes and that males may not always exhibit what is considered to be stereotypical disordered eating behavior (Anderson & Bulik, 2004). For example, the motivation behind weight loss may be a drive for thinness in women, whereas men may strive for more defined muscularity (Murray et al., 2017; Olivardia et al., 2004). Other studies have found that body fat dissatisfaction (i.e., dissatisfaction with body fat percentage), but not muscularity dissatisfaction was associated with disordered eating in men (Smith et al., 2011). A topic on which the literature shows a mixed picture is whether sexual orientation is associated with elevated prevalence of eating disorders or disordered eating in men. Deriving accurate estimates in this population is even more challenging as both topics (i.e., eating disorders and sexual orientation) can be experienced as highly sensitive for men. Homosexual and bisexual men are frequently found to exhibit more signs of ED than heterosexual men, although some studies do no confirm such as association (Bosley, 2011; Calzo et al., 2018; French et al., 1996; Gorrell & Murray, 2019). This may be partly due to the fact that compared to heterosexual male culture, gay male culture places more importance on appearance (Morrison et al., 2004), which could lead more gay men to engage in behavior that place them at risk for developing ED. Harvey and Robinson (2003) showed that gay males had higher body dissatisfaction than heterosexual males, despite being closer to their body ideals.

Finally, given the known impact of ED on quality of life in females (Engel et al., 2009; Le et al., 2019; Winkler et al., 2014), and especially the long-term poor health-related quality of life despite treatment (Pohjolainen et al., 2016), further investigation of quality of life among males with disordered eating is also warranted. A recent study showed that men with ED experience worse decrements in health-related quality of life than women with ED (Hart et al., 2020). However, another study did not find a moderating effect of gender between ED features and health-related quality of life (Wagner et al., 2016). In addition, quality of life seems to be lower among men with muscularity concerns (Tod & Edwards, 2015), and several studies have shown lower quality of life among sexual minorities (Austin et al., 2017; Charlton et al., 2018). To investigate the relationship between quality of life, disordered eating, and drive for muscularity among males, quality of life was also included in the current study.

Accordingly, we conducted an anonymous, online survey using relevant questionnaires (i.e., the Eating Disorder Examination Questionnaire, the Drive for Muscularity Scale, and the Satisfaction with Life Scale) in a large sample of young males aged 15–30 years in Sweden. Our explicit goals were to: (1) investigate whether an anonymous Internet-based survey might be a feasible way of recruiting males and obtaining sensitive information, and (2) assess the nature and magnitude of associations between disordered eating attitudes and behavior, drive for muscularity, satisfaction with life, and sexual preference in males. We hypothesized that the anonymous Internet-based survey would be a feasible recruitment strategy, and that we would find significant positive relationships between disordered eating and drive for muscularity and belonging to sexual minority groups, and that all of these variables would correlate negatively to quality of life.

## Method

### Participants and Procedure

An online Internet survey design was used to recruit Swedish-speaking males between the ages of 15–30 years. The mean age of the participants was 24.79 (SD = 3.64), and their mean body mass index (BMI) was 24.55 kg/m^2^, (SD = 4.74). Potential participants were primarily invited to participate in the study via a link that was disseminated via social media, predominantly Facebook. Upon clicking the link, participants were presented with information detailing the purpose and voluntary nature of the study, giving them the option to quit at any time, and information on how to contact the researchers. A statement of the anonymous nature of the study was provided in an effort to increase the likelihood of participation and candidness of the responses. It was made clear that no personal information that might enable identification would be collected, and that the IP-number would be deleted. Following this information, participants were directed to the survey. As no names or social security numbers were obtained, and the collected data cannot be connected to specific individuals, the study did not need to be approved by any of the Regional Ethical Review Boards in Sweden according to the stipulations concerning the ethical vetting of research on humans that are found in the Ethical Review Act concerning the Ethical Review of Research Involving Humans (2003:460), or the Personal Data Act (1998:204) in Sweden. This was confirmed by the Regional Ethical Review Board in Stockholm that was informed about the nature of the study. Nevertheless, the ethical principles of the Swedish Research Council were followed in every step of the study.

A total of 1817 individuals entered the survey page; however, 687 (37.8%) did not answer any questions. In addition, 178 were excluded based on exclusion criteria (69 were female or did not indicate a gender, 109 did not meet the age criteria of 15–30 years). Of the remaining 1021, 134 did not complete at least one instrument in the survey and were excluded, leaving 824 males that were included in the analyses (i.e., 80.5% of the eligible participants, or 45.3% of those visiting the survey page).

### Instruments

The survey consisted of a total of 85 items. Participants were asked to provide their gender, age, educational background, occupation and living conditions, in addition to completing the following self-report questionnaires.

#### Eating Disorder Examination Questionnaire 6.0 (EDE-Q 6.0)

The EDE-Q (Fairburn, 2008) is a 28-item self-report questionnaire adapted from the EDE (Fairburn & Cooper, 1993) that measures the behavioral and attitudinal features of ED. It provides both a global score as well as four subscale. The psychometric properties of the EDE-Q are generally good, but most studies have been done on female samples. Concurrent validity is satisfactory (Fairburn & Beglin, 1994; Wilfley et al., 1997). The internal consistency (Luce & Crowther, 1999; Mond et al., 2004; Peterson et al., 2007) temporal stability (Luce & Crowther, 1999; Mond et al., 2004) and test–retest reliability of its subscales, and especially the EDE-Q global score (Rose et al., 2013) are all within acceptable ranges. Norms exist for Norwegian males aged 15–30 years old (Reas et al., 2012), Mexican adolescent males (Penelo et al., 2012), American university males aged 18–26 (Darcy et al., 2013) as well as for undergraduate American men (Lavender et al., 2010). Lack of evidence for a solid factor structure of the EDE-Q in non-clinical male samples is a concern, and available suggestions for alternative factor structures for males (e.g., Darcy et al., 2013; Forsen Mantilla et al., 2017) need further replications before they can be employed widely. Consequently, only the EDE-Q global score, and response to behavioral items (e.g., dietary restraint, occurrence of binge eating, and compensatory behaviors) were used in the current study. The internal consistency of the global score of the EDE-Q in this study was 0.93.

#### Drive for Muscularity Scale (DMS)

The DMS is a 15-item measure intended to assess the desire to have increased muscularity in both males and females (McCreary & Sasse, 2000). It has two subscales named the DMS Muscle Development Behaviors and the DMS Muscularity-Oriented Body Image Attitudes. The DMS has been found to have good internal consistency for the full scale (0.87) as well as its subscales (Muscle Development Behaviors = 0.88, and Muscularity-Oriented Body Image Attitude = 0.81), convergent and discriminant validity (McCreary & Sasse, 2000). Cronbach’s alpha in the present study was 0.90 for the full scale, (0.87) for the behavioral, and (0.92) for the attitudinal subscale.

#### Satisfaction with Life Scale (SWLS)

The SWLS is a widely used instrument designed to measure subjectively perceived quality of life and consists of five items (Diener et al., 1985). Each item is scored 1–7 on a Likert response scale (1 = strongly disagree to 7 = strongly agree). Scores on the individual items are summed for a total score where higher scores are indicative of higher satisfaction with life. The SWSL has overall good psychometric properties including high internal consistency (Cronbach’s alpha 0.79 to 0.89; (Adler & Fagley, 2005; Moksnes & Espnes, 2013; Pavot & Diener, 1993; Steger et al., 2006)), and short-term (up to a month) test–retest of 0.8 to 0.83 (Pavot et al., 1991; Steger et al., 2006). The internal consistency of the SWLS in this study was 0.87.

#### Sexual Orientation

The survey also included the question, “How would you describe you sexual orientation?” with four response alternatives: heterosexual, homosexual, bisexual, or other.

### Statistical Analysis

Mean, standard deviation, and median were calculated for age, and all included instruments (i.e., EDE-Q, DMS, and SWSL) as well as their respective subscales when applicable. Frequencies were calculated for demographic variables, sexual preference, and frequency behaviors. The relationship between drive for muscularity and disordered eating attitudes and behavior was, due to the non-normal distribution of the DMS and EDE-Q, calculated using Spearman’s correlation. In order to explore whether eating disordered attitudes and behavior or drive for muscularity differed between groups defined by self-reported sexual preference a Kruskal–Wallis ANOVA was performed. All calculations were performed using IBM SPSS, version 26.

## Results

### Feasibility of Recruitment

A large number of participants could be recruited during a fairly short time frame through simple notes in social media channels (mainly Facebook), although ~ 38% of those who visited the website did not respond to any questions, and 17% were excluded as they did not meet the inclusion criteria. There were no indications of skipping more sensitive questions such as sexual preference, compared to other questions in the survey, which suggests that the anonymous online nature of the study might be helpful for obtaining information on such topics, at least for individuals who click through to the survey and engage with the questions.

### Demographic Characteristics of the Participants

The majority (55.3%) of the participants reported living in a larger city with over 200 000 inhabitants. Over a third (37.4%) reported living by themselves, 26.6% with a partner, 16.4% with their parents, and 14.4% with a friend, and 5% reported other living conditions. More than half of the sample (64.3%) reported having a university education whereas 28% reported completing high school. For current main activity, close to half (49.6%) of the males reported studying, 38% working, 6.8% searching for employment, 1.9% being on long-term or short-term disability, 0.5% being on parental leave, and 2.8% reported another main activity.

### Eating Disorders Psychopathology, Drive for Muscularity and Satisfaction with Life

The participants reported fairly low scores on the indices of eating disorders psychopathology based on the EDE-Q global score. Mean scores, standard deviations, and medians on the EDE-Q, DMS, and SWLS are presented in Table 1.

**Table 1:** Mean, standard deviations and median for the Eating Disorders Examination Questionnaire, Drive for Muscularity Scale and its subscales, and Satisfaction with Life Scale

	Mean (*SD*)	Median (Min–Max)
Eating Disorders Examination Questionnaire: Global score	1.22 (1.07)	0.91 (0–5.25)
Drive for Muscularity Scale: Total score	2.61 (0.99)	2.43 (1–6)
Drive for Muscularity Scale: behaviors	1.83 (0.96)	1.46 (1–6)
Drive for Muscularity Scale: Attitudes	3.39 (1.34)	3.29 (1–6)
Satisfaction with Life Scale	21.89 (6.47)	23.0 (5–35)

Absolut range: EDE-Q Global score: 0–6, Drive for Muscularity Scale (Total and subscales): 1–6, Satisfaction with Life Scale: 1–7

### Core Eating Disorder Behaviors

Around 18% of the males reported that they had engaged in dietary restraint or objective binge eating episodes with around 13% reporting having exercised excessively in the past 28 days. Regular binge eating was the behavior that was most commonly (8%) endorsed. Self-induced vomiting was uncommon and use of laxatives was rare. Table 2 presents a summary of the key disordered eating behavior for the sample.

**Table 2:** Proportion of participants that reported engaging in disordered eating behavior

Key behavior^a^	Any occurrence (%)	Regular occurrence (%)
Fasting	18.3	4.5
Objective binge episodes	18.3	8.0
Self-induced vomiting	1.7	0.5
Laxative misuse	0.5	0.2
Excessive exercise	13.3	2.1

^a^Regular occurrence of fasting was defined as going for long periods of time (8 waking hours or more) without eating anything at all during 13 or more days over the past 28 days, while regular occurrence of objective binge eating, self-induced vomiting or laxative abuse were defined as defined as 4 or more episodes of each behavior over the past 28 days. Regular occurrence of excessive exercise was defined as exercising in a driven or compulsive way to control weight, shape or amount of fat, or burning off calories for ≥ 20 days over the past 28 days

### Relation Among Disordered Eating, Drive for Muscularity, and Satisfaction with Life

We found statistically significant correlations between disordered eating and drive for muscularity (Table 3) in the expected direction. Higher scores on disordered eating measures were significantly correlated with lower satisfaction with life. In addition, the drive for muscularity total score and its attitudinal subscale were also significantly associated with the quality of life, but the relationship between the behavioral aspect of the drive for muscularity and the quality of life was weak and nonsignificant. The two subscales of the DMS correlated moderately with each other.

**Table 3:** Correlations between the eating disorder psychopathology (measured by the global EDE-Q score), disordered eating behaviors (measured by sum of dieting, binge eating and purging items in the EDE-Q), the drive for muscularity (measured by the DMS) and its behavioral and attitudinal subscales, as well as quality of life (measured by the SWLS)

	DMS	DMS behavior	DMS attitudes	SWLS
EDE-Q global	0.43	0.24	0.45	− 0.39
Disordered eating behaviors	0.25	0.18	0.24	− 0.20
DMS		0.73	0.93	− 0.22
DMS behavior			0.45	− 0.03
DMS attitudes				− 0.28

EDE-Q, Eating Disorders Examination Questionnaire; DSM, Drive for Muscularity Scale; SWLS, Satisfaction with Life Scale. All correlations; with the exception of DMS behavior and SWLS were significant at the 0.005 level (2-tailed) after Bonferroni corrections

### Sexual Preference in Relation to Disordered Eating, and Drive for Muscularity

Ten participants (1.2%) did not indicate their sexual preference. Of the remaining 814 participants, the majority, 82% (*n* = 674) described themselves as heterosexual, 9.0% (*n* = 73) as bisexual, 3.4% (*n* = 28) as homosexual, and 4.8% (*n* = 39) as having another sexual preference.

Participants were grouped according to their sexual preferences, and compared. No significant differences emerged across the groups on disordered eating attitudes and behaviors (*H*(3) = 6.44, *p* = 0.09).

In terms of drive for muscularity, the only significant difference between participants according to their sexual preferences was on the DMS behavioral subscale (*H*(3) = 8.38, *p* = 0.04), but the only significant pairwise comparison which was between those endorsing heterosexuality versus those reporting bisexuality failed to remain significant after Bonferroni correction.

## Discussion

This study showed that anonymous Internet survey is a feasible way of recruiting male participants and obtaining sensitive information about topics such as eating disorders and sexual orientation, although the reason for non-engagement of a fairly large subset of individuals who accessed the site is unknown.

As expected, drive for muscularity was positively related to disordered eating attitudes and behaviors. They were both negatively related to satisfaction with life, but the negative association between drive for muscularity and satisfaction with life was mainly due to the attitudinal component of drive for muscularity. We contextualize our results by comparing them with other studies of males in similar age ranges (Table 4).

**Table 4:** Comparison of the Eating Disorders Examination Questionnaire (EDE-Q) global score and key behavior between present study and other studies of males in similar age ranges

Study: Country: Ages: Sample size	Present study Sweden 15–30 (*N* = 824)	Lavender et al USA 18–26 (*N* = 404)	Reas et al Norway 15–30 (*N* = 250)	Penelo et al Spain 18–30 (*N* = 269)	Darcy et al USA 18–26 (*N* = 229)	Cordes et al.^a^ Germany *M* = 29.0 years (*N* = 110)
Measure	Mean (SD)	Mean (SD)	Mean (SD)	Mean (SD)	Mean (SD)	Mean (SD)
EDE-Q Global	1.22 (1.07)	1.09 (1.0)	0.44 (.52)	0.61 (0.70)	0.95 (1.02)	1.01 (0.88)
Key behaviors:	Any occurrence (regular occurrence) % based on EDE-Q data					
Fasting	18.3 (4.5)	24.0 (5.0)	3.2 (0)	9.3 (2.2)	N/A	N/A
Binge episodes^b^	18.4 (8.0)	25.0 (7.9)	9.2 (4)	43.1 (19.0)	N/A	N/A
Vomiting	1.7 (0.5)	3.2 (1.2)	.8 (.4)	0.4 (2.6)	N/A	N/A
Laxative misuse	0.5 (0.2)	2.7 (0.25)	2.4 (0.8)	0.7 (0.4)	N/A	N/A
Excessive exercise	13.3 (1.6)	31.4 (4.5)	28.4 (1.6)	36.4 (30.1)	N/A	N/A

^a^The study by Cordes et al (2021) also provided data on gay males (*n* = 128). Their EDE-Q total score was 1.38 (SD = 1.14)

^b^Binge eating refers to objective binge eating

The global EDE-Q score in the present study is very similar to those found in the US-based studies (Darcy et al., 2013; Lavender et al., 2010), and a recent study from Germany (Cordes et al., 2021), although the US studies collected data from college students who usually score higher on various measures of ED. Higher similarities were expected between the present study and European studies, especially the one done by Reas et al. (2012), which has an identical age span of participants. The difference in the EDE-Q between the present study and the study by Reas et al. (2012) cannot be explained by the anonymous nature of the present study, as participation in the latter study was also anonymous. Nevertheless, the procedure in the Reas et al. (2012) study has not been specified in detail for making a judgment of how truly anonymous the procedure was perceived to the participants. On the other hand, our findings are very similar to a recent study in Germany (Cordes et al., 2021). In that study, the authors also obtained the global EDE-Q values for gay men, which was higher (*M* = 1.38, SD = 1.14) than those reported by heterosexual men (*M* = 1.01, SD = 0.88).

The extent of disordered eating attitudes and behavior as measured by the EDE-Q was similar to findings in a US sample of undergraduate males (Lavender et al., 2010), but higher than what was found in males of approximately the same age from Norway (Reas et al., 2012), Spain (Penelo et al., 2012), and another US sample (Darcy et al., 2013). Note that the Darcy et al. study reported on two samples, one consisting of competitive athletes and one reference group; the comparison made here is to the reference group. In addition, participants in all other studies reported higher rates of excessive exercise compared to the present study, and the regular occurrence of objective binge eating varied from 4 to 19%. However, these discrepancies should be interpreted with caution, given a probable social desirability of reporting high rate of exercise and low reliability of self-report instruments in assessing some of the behaviors such as binge eating.

The similarities of both the eating attitudes and behavior measured by the EDE-Q and frequencies of key ED behaviors to the US sample collected by Lavender et al. (2010), with a large sample adds credibility to our findings, but the differences compared to the Norwegian and Spanish studies are noteworthy. The higher EDE-Q-total values in our study might be related to the true anonymous nature of our study. It cannot be explained by higher BMI, and potentially not higher drive for muscularity compared to other studies.

Levels of drive for muscularity, as measured by the DMS, were in-line with results based on a sample of approximately 300 Canadian males attending high school or university (McCreary et al., 2004), with mean on the total score, the attitudinal subscale and the behavioral subscale being 2.61, 3.39, 1.83 in the present study, and 2.69, 3.5 and 2.06 in the study by McCreary et al. (2004). The mean total score of DSM in a recent online, non-anonymous study by Cordes et al. (2021) for heterosexual male was 2.70, and 2.69 for the gay subsample. The similarities are striking, especially considering the difference in study procedures (i.e., complete anonymity in the present study versus no anonymity in the other two studies). This might suggest that young males are prone to provide an accurate picture of their drive for muscularity irrespective of the level of anonymity in the surveys.

Satisfaction with life, as measured by the SWLS, was somewhat lower in the present sample than what has recently been reported among Norwegian adolescents (Moksnes & Espnes, 2013) and the US undergraduates (McFarland & Petrie, 2012). Interestingly, we found weak, but significant negative correlations between the DMS and its attitudinal subscale to the SWLS, but the behavioral subscale of the DMS was not significantly related to the SWLS. It is expected to see a somewhat lower satisfaction with life among people high on DSM attitudes. However, those who engage in DMS behavior might be high or low on SWLS as some of them might be content with engaging in behavior that is in-line with their values (being muscular), while others might find their engagement to be not enough to help them achieve their goals. This pattern might explain the moderate correlation between the subscales of the DMS and their relation to the SWLS, but future studies need to empirically test this assumption by investigating whether outcome expectancies might moderate the relationship between DMS subscales and SWLS.

Finally, with reference to self-reported sexual preference, the majority reported a heterosexual preference, followed by bisexual preference, other sexual preferences, and finally homosexual preference. Although the percentage of homosexual preference is in-line with data of sexual behavior in the US documented by Seidman and Rieder (1994), the percentages of both homo- and bisexual preferences in the present study are higher than that reported by Chandra et al. (2011) in a US National survey from 2006 to 2008. Although young males who endorsed homo-, or bisexuality reported numerically higher EDE-Q global scores than those who reported heterosexuality, the differences were not significant. This finding diverges from previous research (Bosley, 2011). The mixed findings might be a consequence of operationalization of sexual preference, or true difference in the samples. While 41% of men in the study by Yean et al. (2013) reported being exclusively gay (no overlapping with bisexuality or other orientations), the corresponding figure in the present study was 3.4%. The inconsistent findings justify further investigations to identify the variables that drive such an association in some samples and not others, leading to mixed findings.

The results should be interpreted in the light of the limitations and strengths of the study. The educational level of the study sample was much higher than that of the total population of males 16 years of age or older in Sweden, where only 29% have a university education and 45% have completed high school. However, if we take into consideration that a much higher proportion of the participants in the present study live in a large city, it may be more relevant to compare the sample with the male population living in larger urban centers. Here we find that for males 16 years or older, 43% have a university education and 36% have completed high school. Thus, the sample seems fairly representative of the population of young males in larger cities. Slightly higher education level of those who choose to participate in academic surveys, or more specifically the inverted-U relation between survey compliance and income, is a common phenomenon, as discussed at book length by for example Korinek et al. (2005).

The use of the EDE-Q for assessment of disordered eating attitudes and behavior in males may not be as accurate as for females. Much of the past research on the EDE-Q is based on female participants, and the psychometric properties of the EDE-Q in clinical and non-clinical male samples needs further investigation. It is known that males may not always exhibit stereotypical disordered eating behavior (Anderson & Bulik, 2004). Regardless, the EDE-Q is widely used by researchers and clinicians when assessing both males and females. In order to capture the male presentation of disordered eating attitudes and behaviors, the use of the EDE-Q was combined with the Drive for Muscularity Scale in the present study. Despite the potential limitations of the EDE-Q with males, its use here enabled comparisons to normative data collected on males in other studies, as shown above.

Another limitation is the fact that using an opt-in Internet questionnaire to gather data provides a non-probability sample which may be limited in its generalizability to the population. However, nonprobability samples are judged to be acceptable for use in preliminary, exploratory studies (Statistics Canada). Additionally, the sampling method used offered the advantages of collecting a sufficiently large sample to explore relationships between, for example, sexual preference and disordered eating as well as likely contributing to more candid responses to questions with potentially sensitive nature. As a significant portion of the respondents live in one of the largest cities in Sweden the generalizability of findings to those living in rural areas might be limited.

Finally, the assessment of sexual preference based on a single question with mutually exclusive answers may not accurately capture the complexity of sexual preferences, and assessment of disordered eating based on self-report only presents another methodological limitation.

To conclude, anonymous Internet survey seems to be a feasible way of recruiting male participants and obtaining sensitive information about eating disorders and sexual orientation—both of which can be perceived as sensitive and stigmatized topics. Drive for muscularity was positively related to disordered eating attitudes and behaviors. The latter and the attitudinal component of the drive for muscularity were negatively related to satisfaction with life among young males. Sexual orientation was not associated with disordered eating attitudes or behaviors. Mixed findings, different definitions and categorization of sexual orientation, and the sensitive nature of questions regarding sexuality and ED among men justifies further anonymous surveys with markedly larger sample sizes across different countries using the same definitions and research procedures to resolve inconsistencies enabling more accurate characterization of the nature of EDs in males.

## Data Availability

Data are available upon request from the corresponding author.
